# Shift of bacterial community structure along different coastal reclamation histories in Jiangsu, Eastern China

**DOI:** 10.1038/s41598-017-10608-3

**Published:** 2017-08-30

**Authors:** Jianfeng Hua, Youzhi Feng, Qian Jiang, Xuewen Bao, Yunlong Yin

**Affiliations:** 1 Institute of Botany, Jiangsu Province and Chinese Academy of Sciences, Nanjing, China; 2 State Key Laboratory of Soil and Sustainable Agriculture, Institute of Soil Science, Chinese Academy of Sciences, Nanjing, China; 3 Nanjing Forest Police College, Nanjing, China

## Abstract

Tideland reclamation has drastic effects on coastal ecosystem involved in soil microorganisms. However, the knowledge regarding temporal variations of microbial community along reclamation chronosequence and their environmental variable predictor is still poorly known. Using Illumina sequencing, we qualified bacterial community composition in soils collected from one tideland and four reclamation stages, *i.e*. 2-year, 7-year, 19-year and 39-year in Jiangsu, Eastern China. Across all samples, the dominant groups were Proteobacteria, Bacteroidete, Acidobacteria, Planctomycetes and Chloroflexi. Reclamation activity and its histories greatly altered bacterial community structure, and only 0.28% of phylotypes were shared by five soils. Specially, some typical marine bacteria (*Gaetulibacter*, *Alcanivorax* …) disappeared in reclamation soils, while other groups (*Niabella*, *Flavisolibacter*…) were gradually eminent. Generally, bacterial diversity and richness increased with reclamation histories. Bacterial community was correlated with most of soil physico-chemical properties. Amongst, mean weight diameter of soil aggregates (MWD) was detected as a primary factor predicting bacterial community composition. Together, our results indicated that effects of reclamation on bacterial community varied with diked histories, and MWD was a major factor predicting bacterial community during progressive reclamation. These findings offer predicting case study for understanding the impact of reclamation and its histories on microbial community in a coastal ecosystem.

## Introduction

The coastal tideland is an interface between the ocean and land. It is an important wetland ecosystem characterized by frequent exchange and transformation of materials and energy^1^. Meantime, tideland is a vital land source for agricultural production and urban development in coastal areas^2^. In order to relieve population pressure, ensure food safety and promote regional economy, reclamation from tidelands has become a foundational strategy in many countries such as South Korea^3^, Netherlands^4^ and Malaysia^5^. In particular, coastal reclaimed lands are large and steadily increasing in China^6, 7^. Taking the example of Jiangsu Province which has the largest prograding mudflat in Asia^8^, more than 0.23 million ha tidelands has been reclaimed over the last couple of years^9^. Moreover, additional 0.18 million ha tidelands would be reclaimed from 2010 to 2020 according to *the Jiangsu Beach Reclamation Development Planning Outline (2010–2020)* ^10^.

Microorganisms are abundant and diverse in soils, and they play important roles in nutrient cycling and stability of ecosystems. As one of soil component, microbial community is strongly shaped by soil characteristics, especially the bacteria which were the most abundant group of microorganisms. At present, some studies have revealed that pH is a critical factor shaping the bacterial community structure in agriculture filed^11^, forest and grassland^12^ and even Arctic^13^. Also, it could be influenced by soil type^14^, texture^15^ and nitrogen availability^11, 16^. Coastal land reclamation has been well demonstrated to have profound impacts on soil properties, such as pH^17^, electrical conductivity^7^ and organic carbon^18^. As a result, bacterial community could be affected greatly. For example, some bacteria typically found in marine and saline environments disappeared from reclamation soil^19^. Notably, the effects of reclamation on soil properties differed by the different length of time following reclamation^20, 21^. Most soil characters changed a lot in the initial reclamation stages. A relatively steady state was reached within 10 years following the reclamation for pH, about 30 years for organic matter, and 60 years for electrical conductivity, respectively^7^. Here, we can see that differences in diked histories would induce different soil properties, and thus different influences on microorganisms^19^. Cui *et al*.^17^ found that abundance of *Acaulospora* decreased with reclamation years and compared to 2-year reclamation land, both 6-year and 34-year sites had lower species richness and dominance of arbuscular mycorrhizal fungi. However, little information is available on the effects of tideland reclamation histories on soil bacterial structure and diversity.

Conversion of tideland to terrestrial ecosystems by dike provides an ideal system for understanding the variability in bacterial community structure along the time following reclamation as well as its mechanism. Such study would provide important scientific bases for sustainable land use in coastal areas. In this investigation, five soils, one tideland and four reclaimed lands with different histories represented as 2-year, 7-year, 19-year and 39-year, were sampled in coastal Jiangsu, Eastern China. Here, we hypothesized that along the reclamation histories, bacterial community composition is varied distinctly owing to the changes of soil physic-chemical properties. The objectives of this study were to compare the abundance, composition and diversity of bacterial community among the five soils through Illumina sequencing, and to explore the contributions of environmental variables that correlated to changes in the structure of bacterial community.

## Results

### Soil physico-chemical properties

The distribution of soil aggregates extensively varied among five sites. It was found that silt + clay fractions (<0.053 mm) (SC) represented the greatest fraction of whole soil for native, 2-year and 7-year which were 94.1%, 90.0% and 76.5%, respectively, but large aggregates (>1.0 mm) (LA) were not observed in these sites (Table 1). With the increasing reclamation histories, the amounts of aggregates (>0.053 mm) were increased. Especially, LA was progressively formed at 19-year and 39-year. However, SC was declined by 18.7, 43.3 and 57.6% at 7-year, 19-year and 39-year, respectively compared to native. These trends indicated that larger aggregates are formed of silt, clay or individual particles during the progressive reclamation. Consequently, mean weight diameter of soil aggregates (MWD) was 0.81, 3.02 and 3.60 fold greater (*P* < 0.05) at 7-year, 19-year and 39-year, respectively than that at native. Soil bulk density (BD) was decreased with increasing reclaimed years, and significant difference (*P* < 0.05) was observed between native and 39-year (Table 1).

**Table 1:** Soil physical properties at five sampling sites.

Sites	BD (g cm^−3^)	MWD (mm)	Aggregate size distribution (%)			
			LA	MAA	MIA	SC
Native	1.42 ± 0.06a	3.51 ± 0.03d	—	0.26 ± 0.17c	5.66 ± 1.05c	94.1 ± 0.88a
2-year	1.37 ± 0.05ab	4.52 ± 0.81cd	—	1.32 ± 1.19c	8.66 ± 2.25bc	90.0 ± 2.71ab
7-year	1.36 ± 0.01ab	6.37 ± 0.87c	—	1.65 ± 1.03bc	21.9 ± 2.52b	76.5 ± 3.32b
19-year	1.33 ± 0.10ab	14.1 ± 2.33b	1.80 ± 0.29a	3.02 ± 0.92b	41.8 ± 15.51a	53.4 ± 15.5c
39-year	1.29 ± 0.04b	16.4 ± 0.77a	0.85 ± 0.38b	9.47 ± 0.25a	49.7 ± 5.26a	39.9 ± 4.93c

Data presented are means ± standard deviation (*n* = 3). Means followed by the different letters into each column are significantly different according to Duncan’s Multiple Range Test at 5% level. BD, soil bulk density; MWD, mean weight diameter of soil aggregates; LA, large aggregates (>1.0 mm); MAA, macroaggregates (1.0–0.25 mm), MIA, microaggregates (0.25–0.053 mm), SC, silt + clay fractions (<0.053 mm). —, not observed.

Soil pH ranged from 8.39 to 9.08, and 7-year had the highest value (*P* < 0.05). OM and AP were much greater (*P* < 0.05) at 19-year than those at other soils (Table 2). TN varied from 0.02% to 0.12%, and the higher (*P* < 0.05) values were found at 19-year and 39-year (Table 2). EC and soluble salt ions were remarkably decreased along the reclamation years except for CO_3_^2−^ and HCO_3_^−^. Compared to native, significant reduction (*P* < 0.05) of EC (89.1–98.9%) and Mg^2+^ (95.4–98.9%) at 7-year, 19-year and 39-year, and Na^+^ (99.7%), Cl^−^ (99.8%) and SO_4_^2−^ (98.9–99.1%) at 19-year and 39-year were found, respectively. Likewise, decreased (*P* < 0.05) Ca^2+^ and K^+^ were observed at all four reclamation sites, and the lowest values were recorded at 7-year and 39-year which were decreased by 89.6% and 89.8% over native tideland, respectively. Noticeably, for most of the tested physico-chemical properties, no significant difference was observed between native and 2-year.

**Table 2:** Soil chemical properties at five sampling sites.

Sites	pH	TN (%)	OM (%)	C:N	AP (mg 100 g^−1^)	EC (ms cm^−1^)	Ca^2+^ (mg kg^−1^)	K^+^ (mg kg^−1^)	Mg^2+^ (mg kg^−1^)	Na^+^ (mg kg^−1^)	Cl^−^ (mg kg^−1^)	SO_4_^2−^ (mg kg^−1^)	CO_3_^2−^ (mg kg^−1^)	HCO_3_^−^ (mg kg^−1^)
Native	8.39 ± 0.07b	0.03 ± 0.00 cd	0.49 ± 0.05b	8.18 ± 0.65a	0.62 ± 0.04b	7.22 ± 1.43a	302 ± 100a	268 ± 24.6a	438 ± 160a	2955 ± 1337a	6079 ± 3481a	1276 ± 731a	0.00 ± 0.00b	123 ± 11.9c
2-year	8.55 ± 0.21b	0.02 ± 0.00d	0.26 ± 0.05b	7.68 ± 0.42a	0.41 ± 0.09b	5.18 ± 3.40a	166 ± 107b	185 ± 75.1b	338 ± 343a	3008 ± 2385a	5922 ± 5754a	891 ± 661ab	4.56 ± 1.32b	122 ± 29.7c
7-year	9.08 ± 0.28a	0.04 ± 0.01c	0.35 ± 0.01b	4.94 ± 0.96b	0.34 ± 0.09b	0.79 ± 0.49b	31.3 ± 1.16 c	62.8 ± 11.4c	20.3 ± 0.63b	809 ± 619ab	782 ± 682ab	219 ± 193bc	36.9 ± 8.97a	353 ± 47.2a
19-year	8.44 ± 0.06b	0.12 ± 0.03a	1.49 ± 0.48a	6.90 ± 1.11a	3.81 ± 2.63a	0.11 ± 0.03b	56.9 ± 5.33bc	87.6 ± 35.2c	9.02 ± 0.50b	8.85 ± 1.53b	10.7 ± 1.98b	13.4 ± 2.23c	0.00 ± 0.00b	220 ± 20.9b
39-year	8.48 ± 0.11b	0.08 ± 0.00b	0.68 ± 0.06b	4.89 ± 0.38b	0.30 ± 0.08b	0.08 ± 0.02b	60.9 ± 10.4bc	27.4 ± 9.87c	4.83 ± 1.05b	6.83 ± 2.76b	12.3 ± 5.20b	11.1 ± 5.18c	0.00 ± 0.00b	166 ± 2.84c

Data presented are means ± standard deviation (*n* = 3). Means followed by the different letters into each column are significantly different according to Duncan’s Multiple Range Test at 5% level. TN, total nitrogen; OM, organic matter; AP, available phosphorus; EC, electric conductivity.

### Structure and diversity of soil bacterial communities

**Figure 1:**
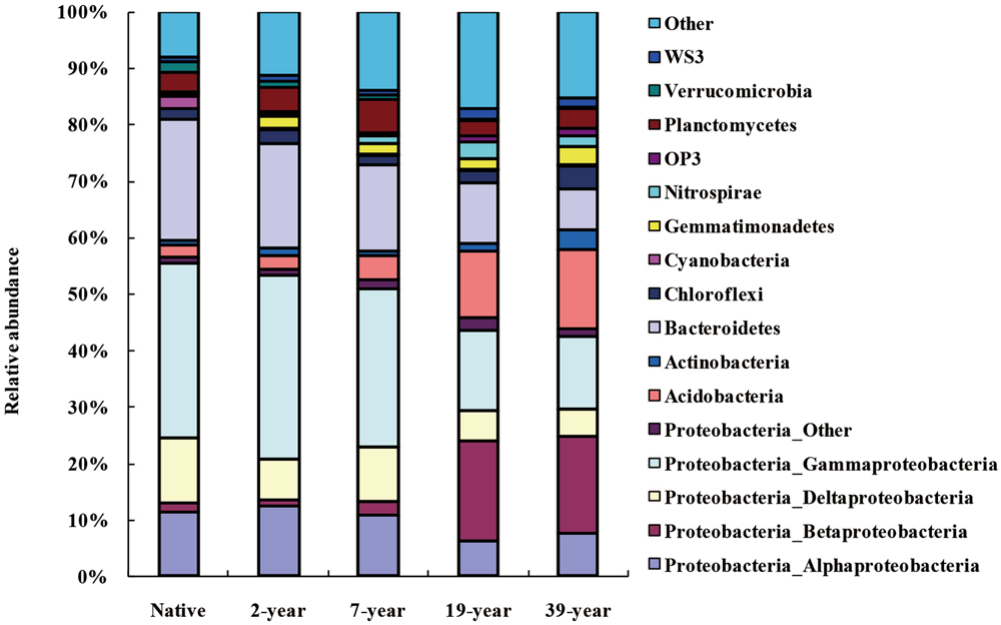
A 100% stacked column chart of relative abundances of the dominant bacterial phyla (proteobacterial class) derived from 16 S rRNA genes at five sampling sites.

After filtering the sequence reads by base quality and removing reads smaller than 200 bases, we obtained a total of 281,649 high-quality 16 S rRNA gene sequences from the five soil samples. The number of high quality sequences per sample varied from 7,604 to 24,650 and the average number of sequences per sample was 18,776. Across all samples, 127,238 (74.83%) were classified below the domain level when grouped at the 97% similarity level. The classified sequences were affiliated to 12 bacterial phyla across all sites. The five dominant phyla (among all obtained sequences) were Proteobacteria (50.65%), Bacteroidetes (14.70%), Acidobacteria (6.89%), Planctomycetes (3.94%) and Chloroflexi (2.48%). In addition, seven phyla (Gemmatimonadetes, Actinobacteria, Nitrospirae, WS3, Verrucomicrobia, OP3, Cyanobacteria) were considered low abundant with sequence frequencies below 2% (Fig. 1).

**Figure 2:**
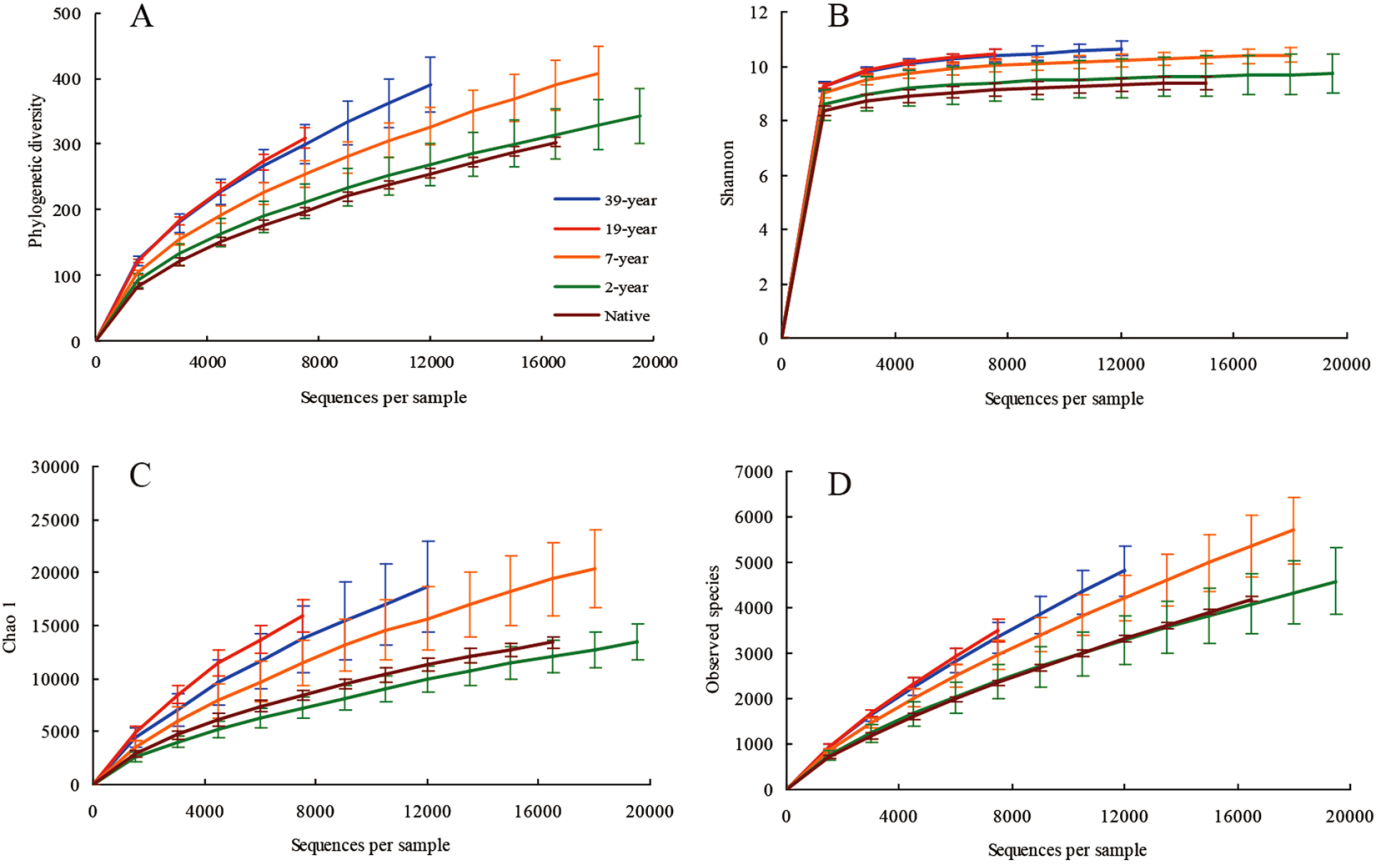
Rarefaction curves of Phylogenetic Diversity index (**A**), Shannon index (**B**), Chao1 (**C**), and observed species (**D**) at five sampling sites.

In the present investigation, bacterial diversity (phylogenetic diversity and Shannon) and richness (Chao1 and observed species) showed a potentially increasing trend along the reclamation histories and no differences were found between any two adjacent sites except for Chao 1 (Fig. 2). However, statistical differences (*P* < 0.05) in phylogenetic diversity, Shannon Chao1 and observed species were observed between native and 2-year lands, and 19-year and 39-year soils. In this study, 7-year site acted like a bridge between native and the newly reclaimed sites and the long-term reclaimed lands.

**Figure 3:**
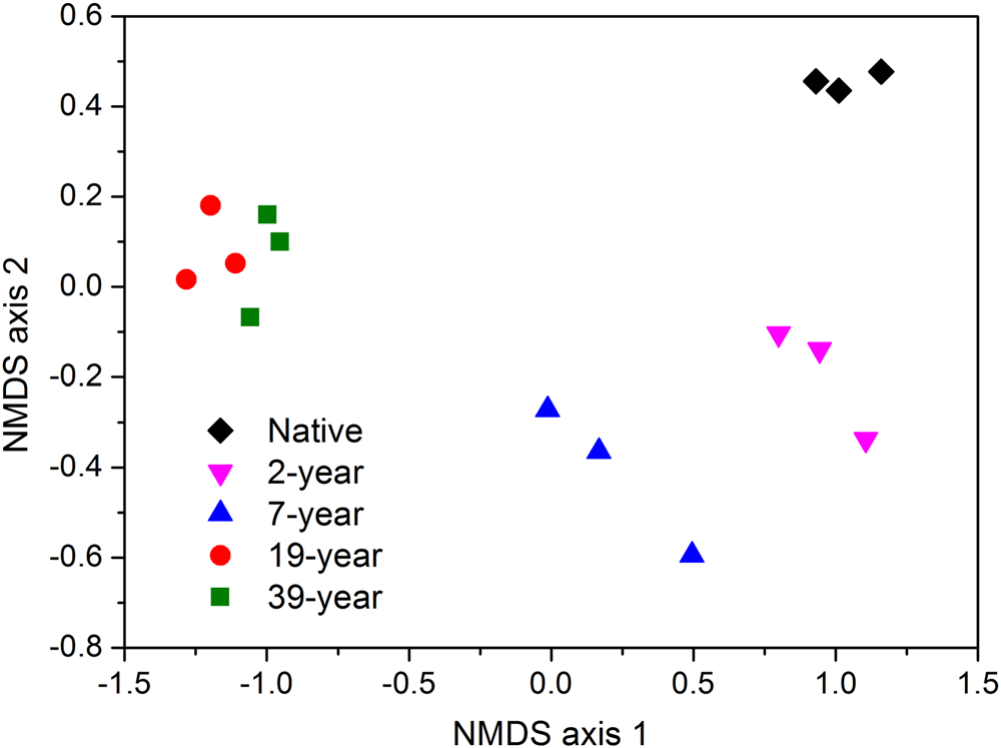
Bacterial community compositional structure in soils as indicated by a non-metric multi-dimensional scaling plot of the weighted pairwise UniFrac community distances between different soil samples.

Significant shifts of soil bacterial community were found according to a NMDS plot (Fig. 3). Native, 2-year, 7-year and the cohesive group of 19-year and 39-year were well separated. The distances between tideland and other samples along the NMDS axis 1 further implied that the effects of reclamation on soil bacterial community increased along the reclamation histories.

### Distinct bacterial groups

Five sites shared 13 phylotypes (0.28% of total phylotypes), and 75.54% of phylotypes were detected in a single site at the 97% OTU cut-off value (see Supplementary Fig. S1). The highest (836) and lowest (497) numbers of unique phylotypes were found in soils of 39-year and 2-year, respectively. The number of phylotypes shared by any two sites was between 3 and 476. The highest number of shared OTUs was found in 39-year and 19-year sites, and the lowest was observed in 39-year and native sites, and 39-year and 2-year sites, respectively. Additionally, the numbers of shared OTUs between the native and four reclaimed sites sharply reduced along the reclamation histories, which were 207, 31, 4, and 3, respectively (see Supplementary Fig. S1).

**Figure 4:**
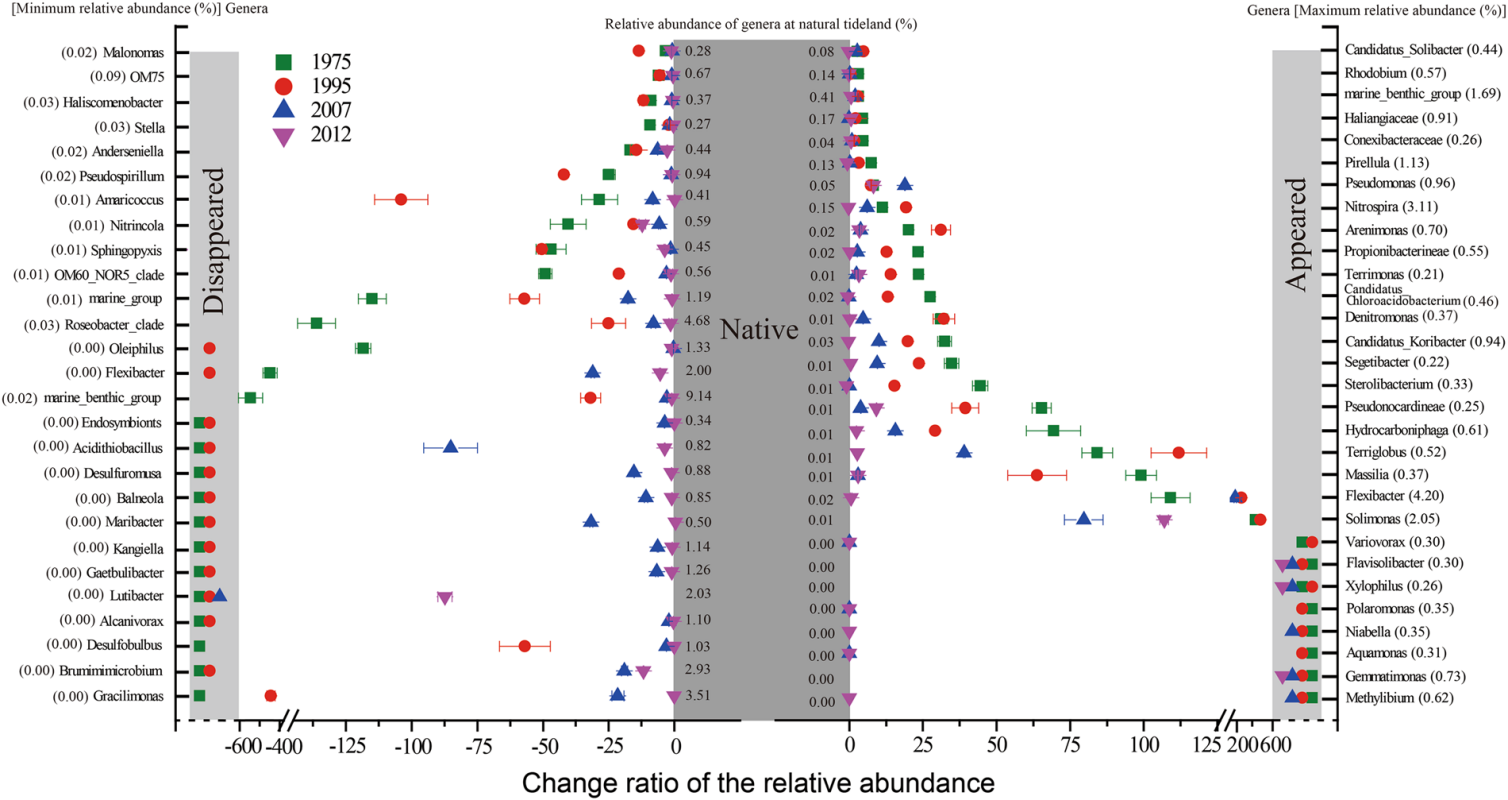
Change ratio of the relative abundance of dominant genera in four reclaimed soils relative to native tideland soil.

There were also significant differences in the relative abundance of certain bacterial groups at lower taxonomic levels. Change ratios of the dominant genera were calculated based on the relative abundance in four reclaimed soils relative to tideland soil (Fig. 4). Compared to native soil, the relative abundances of 27 dominant genera were declined along the reclamation year. Of note, 9 genera, *Endosymbionts*, *Acidithiobacillus*, *Desulfuromusa*, *Balneola*, *Maribacter*, *Kangiella*, *Gaetbulibacter*, *Lutibacter*, *Alcanivorax* and *Brumimimicrobium*, the percentages ranging from 0.34% to 2.03% at native soil, did not appear at 19-year and 39-year soils. By contrast, the percentages of 28 dominant genera were sharply increased along the reclamation histories when compared with native soil (Fig. 4). Among them, 8 genera, *Variovorax*, *Flavisolibacter*, *Xylophilus*, *Polaromonas*, *Niabella*, *Aquamonas*, *Gemmatimonas* and *Methylibium* were absent at native soil, but normally detected at reclaimed soils.

### Correlation analysis of bacterial communities against soil properties

Bacterial diversity and richness were positively (*P* < 0.05) correlated with MWD, LA, MAA, MIA, TN, OM and HCO_3_^−^, but negatively (*P* < 0.05) with SC, EC, C:N ratio, Ca^2+^, K^+^, Mg^2+^, Na^+^, Cl^−^ and SO_4_^2−^. With regard to the relative abundance of phyla (proteobactrial classes), they were also highly correlated with soil variables. For example, the relative abundance of Acidobacteria was positively (*P* < 0.05) correlated with MWD, LA, MAA, MIA, TN, OM and HCO_3_^−^, and negatively (*P* < 0.05) correlated with SC, C:N ratio, EC, Ca^2+^, K^+^, Mg^2+^, Na^+^, Cl^−^ and SO_4_^2−^. However, the relative abundance of Bacteroidetes showed an opposite pattern (*P* < 0.05). Within the phyla Proteobacteria, the relative abundance of Gammaproteobacteria, Deltaproteobacteria and Alphaproteobacteria were positively (*P* < 0.05) correlated with SC, EC, Mg^2+^, Na^+^, Cl^−^, SO_4_^2−^, and negatively (*P* < 0.05) related with MWD, LA, MAA, MIA and TN, while an opposite trend was observed for Betaproteobacteria (*P* < 0.05) (see Supplementary Table S1).

The relative abundances of the distinct genera whose amounts were decreased with reclamation histories were negatively correlated with MWD, LA, MAA, MIA and TN (*P* < 0.01), while positively related to SC, EC, K^+^, Mg^2+^, Na^+^, CL^−^ and SO_4_^2−^ (*P* < 0.01). However, for those groups whose relative abundances were enhanced with reclamation histories, the opposite correlated patterns were observed (*P* < 0.01) (see Supplementary Table S2). Moreover, pH was only related to the relative abundance of Cyanobacteria (*P* < 0.05), and no significant correlation between AP and bacterial community was observed.

**Figure 5:**
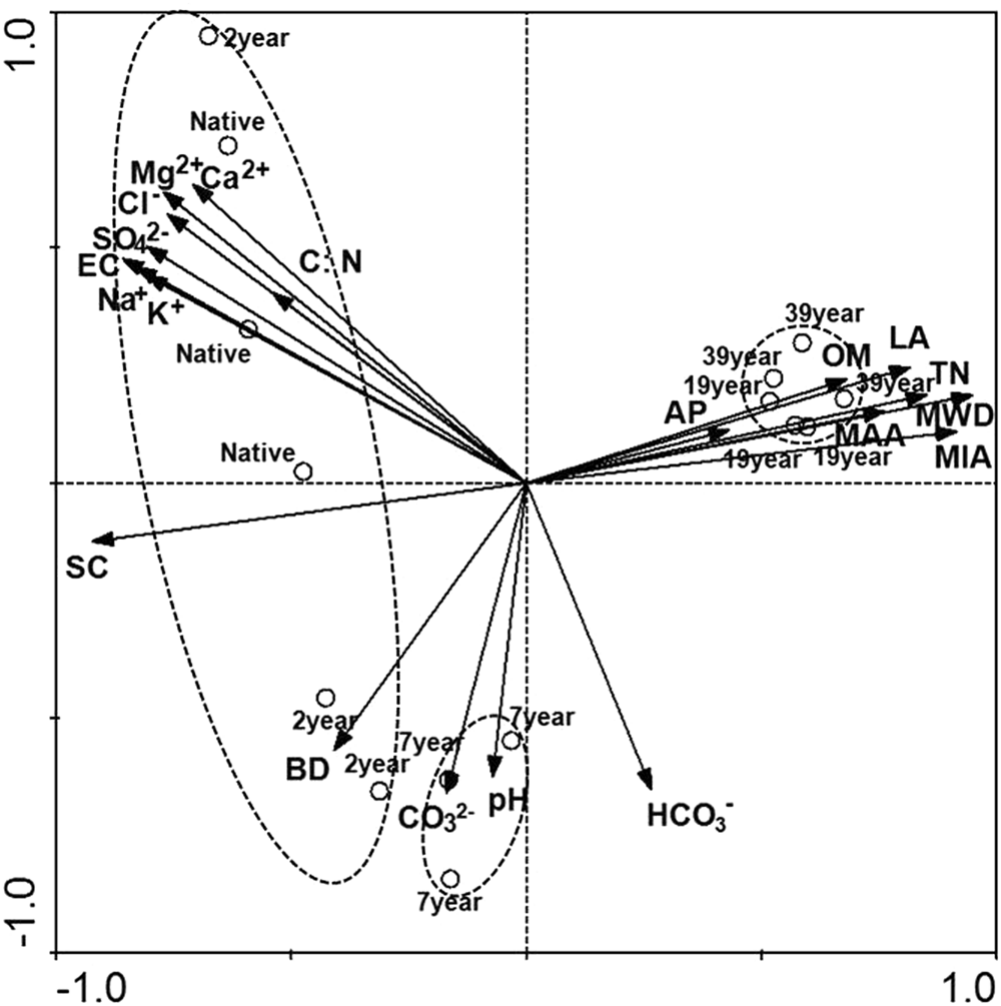
Redundancy analysis (RDA) of bacterial community composition and soil variables for individual samples.

Results of the Mantel test indicated a remarkable positive correlation (*P* < 0.05) between bacterial community structure – more specifically, the Bray-Curtise distance – and all soil properties except for AP. Table 3 listed the environmental factors from highest to lowest Spearman’s correlation scores. Intriguingly, soil physical properties MWD, SC and MIA possessed the top three followed by EC and TN, while pH and AP ranked last two. RDA analysis also demonstrated a strong relationship between bacterial community structure and soil properties in the studied sites (Fig. 5). The first two axes of RDA explain 55.8% and 17.3%, respectively, of the total variation in the data. Bacterial communities of 19-year and 39-year sites were more alike and related to higher MWD, MIA and TN, as showed by their close grouping and by the vectors. On the other hand, bacterial communities of native land and 2-year site formed a separate group associated with higher EC and SC, while the bacterial community of 7-year site separated from others with higher CO_3_^2−^ and HCO_3_^−^.

**Table 3:** The spearman’s correlations (*r*) between soil properties and bacterial community structure (Braye-Curtis distance) determined by Mantel test.

Soil properties	*r*	*P*
MWD	0.709	0.001
SC	0.640	0.001
MIA	0.626	0.001
EC	0.557	0.001
TN	0.514	0.001
Na^+^	0.498	0.001
K^+^	0.492	0.001
LA	0.455	0.001
SO_4_^2−^	0.447	0.001
Mg^2+^	0.416	0.001
Ca^2+^	0.401	0.001
Cl^−^	0.397	0.001
HCO_3_^−^	0.367	0.001
BD	0.282	0.013
MAA	0.279	0.005
CO_3_^2−^	0.278	0.001
OM	0.273	0.001
C:N	0.250	0.018
pH	0.204	0.008
AP	0.035	0.267

BD, soil bulk density; MWD, mean weight diameter of soil aggregates; LA, large aggregates (>1.0 mm); MAA, macroaggregates (1.0–0.25 mm); MIA, microaggregates (0.25–0.053 mm); SC, silt + clay fractions (<0.053 mm); TN, total nitrogen; OM, organic matter; AP, available phosphorus; EC, electric conductivity.

According to a distance-based regression analysis, MWD was significantly related to bacterial community composition (*P* < 0.001). It explained 25.44% of variation in bacterial community structure (Table 4). Together, the data strongly implicate that the shifts of bacterial community composition were mainly mediated through the alteration of MWD followed by TN and EC.

**Table 4:** Relationship of soil variables with the bacterial community composition as revealed from distance-based regression analysis.

Variance explained (%)	*P* value	Variables in model
25.44	<0.001	MWD
12.63	<0.001	CO_3_^2−^

MWD, mean weight diameter of soil aggregates.

## Discussion

### Responses of bacterial community structure

All of the bacterial communities in investigated soils were dominated by phyla Proteobacteria, Bacteroidetes and Acidobacteria. This observation was consistent with the findings in forest^22^, agriculture^23^ and Arctic^13^. However, significant differences in bacterial community composition were found among the five soils in this study. In particular, the relative abundance of Acidobacteria was increased, while those of Bacteroidetes and Proteobacteria were reduced along the years following reclamation. As previously reported, Acidobacteria was specially adapted to acidic and low level of nutrients soils^24, 25^. In this study, however, its abundance was negligibly related to pH and positively correlated to TN and OM. In fact, most of the subdivisions of Acidobacteria are unculturable and only known by 16 S rRNA gene sequences, and their physiology and ecological functions remain unclear^26^. Considering the presence and abundance, Acidobacteria may play an essential role in ecological functions of coastal soils and other soil types^24^. Proteobacteria presented a constant most abundance (56.61–43.97%) in all soils, apparently indicating that this phylum may not be largely affected by reclamation. Actually, Proteobacteria is a broad phylum of the bacteria domain which includes a great level of morphological, physiological, and metabolic diversity^27^. Thus, when the entire phylum is taken into account, particular differences in some subgroups may not be observed. In the present study, it was found that different subgroups of this phylum were differently affected by reclamation histories. The proportion of Alphaproteobacteria, Deltaproteobacteria and Gammaproteobacteria shared a similar pattern with Proteobacteria. Besides, negative correlations between them and TN and OM were observed. However, the relative abundance of Betaproteobacteria showed an opposite trend which was increased with the histories, and was positively correlated with TN and OM. As a copiotrophic bacteria (fast growing), Betaproteobacteria generally was considered as an indicator of soils with high nutrients^28^. Obviously, long-term reclaimed sites enriched with greater nutrients such as TN and OM, benefit the growth of Betaproteobacteria, but hinder the propagation of Deltaproteobacteria and Gammaproteobacteria which are mostly characterized as chemoautotrophic bacteria^29^.

Microbial diversity is critical to the integrity, function, and long-term sustainability of soil ecosystems^30^. In the present investigation, bacterial diversity and richness were increased by reclamation, and generally, the positive effects were enhanced along the years following reclamation. This finding indicated that tideland reclamation would benefit the stability of microbial functions and soil ecosystem^31^. Among all five sites, bacterial diversity and richness as well as the abundance of most bacterial phyla were intermediate at 7-year site, which suggested the markedly developing role of medium-reclaimed land in the coastal ecosystem. The two sites – 19-year and 39-year– shared the similar patterns of bacterial diversity and richness and even composition. It means that bacterial community structure at reclaimed soils will keep at a stable level after a long-term acclimation and evolution. Meanwhile, most of soil physico-chemical properties, after 19 years of reclamation, were significantly differed from other investigated sites. Here, we can find that coastal reclamation has positive effects on soil ecosystem, and after nearly 20 years, reclaimed lands will have a more stable soil ecosystem, which was in accordance with the outcome of Guo *et al*.^32^.

### Contribution of environmental factors to bacterial community structure

Previous studies have successfully documented that both physical and chemical properties could be greatly altered from a near-marine environment to a terrestrial one because of the dikes, and differences in the length of time following reclamation would introduce different effects^7, 21, 33^. Also, larger aggregates, increased nutrients and reduced EC were found during the progressive tideland reclamation in the present study. Definitely, the variation of soil properties would affect soil bacterial community structure. As we expected, most of tested soil physical and chemical properties were significantly correlated with bacterial community composition. Amongst, MWD which is an index of soil aggregate stability was detected as a main factor shaping bacterial community.

Recently, soil pH has been detected as the most predominant factor in various soils^11, 23, 34^, but there are also exceptions^29, 35, 36^ as well as present experiment. Of note, soils investigated in previous studies usually were neutral or less acidic samples, and had large variation in pH, while minimal differences in other parameters^34, 37^. In this study, however, coastal alkaline soils were investigated and a relatively small pH range (8.39–9.08) was observed. Furthermore, other soil physico-chemical properties substantially varied among five sites, which were in accordance with other studies in coastal reclamation areas^7, 33^. For example, EC was decreased by 99.8% while MWD was increased by 367% at 39-year site when compared with tideland. Together, pH may play a negligible role in determining bacterial community in soils where the other properties vary more than pH^35, 36^.

From the current literatures, we found that compared with soil chemical parameters, less works concentrated to physical properties when analyzing the driving factors of microbiological community^13, 36, 38^. However, physical properties such as water content, nature of aggregation and porosity shared the important functions with chemical parameters in structuring the bacterial community^14, 15, 22^. In this study, soil aggregate size distribution which is a crucial aspect of ecosystem functioning in terrestrial ecosystems^39^, sharply changed along the reclamation years. Larger aggregates were formed of silt, clay or individual particles during the progressive reclamation. Meanwhile, MWD was significantly correlated with bacterial diversity and richness as well as the relative abundance of most taxonomy. Consistently, the results in grassland restoration soils revealed that MWD was highly correlated with total PLFA biomass and the biomass of many microbial groups^40^. Our results support the idea that distribution pattern of microbial biomass and activity are governed by aggregate size in some specific situations^41, 42^. This phenomenon could partly be explained by that soil aggregation directly affects various soil physical, chemical and biological processes, such as soil aeration and soil water infiltration, and then leads to the heterogeneous distribution of microbes among aggregates of different sizes^42, 43^. For instance, larger aggregation (1.0–2.0 mm) had the highest levels of soil organic C, microbial biomass C and soil respiration among all tested aggregates^44^. Indirectly, aggregate fractions could significantly affect the composition of microbivores, such as soil nematodes which are one of the most abundant groups of soil invertebrates and feeding on microbes, and consequently, shift the microbial biomass, diversity and activity^41, 45^. Hence, it would be interesting to investigate the shift of bacterial community in soil aggregates with different size during the progressive land reclamation in coastal areas.

### Distinct bacterial groups

Only 0.28% of phylotypes were shared by five soils and quite a large proportion (49.4–66.1%) of phylotypes were unique to each site in this study. The average value of unique phylotypes (58.1%) was greater than the result (48.6%) observed in alder stands soil^22^, but was lower than that (75.0%) reported in a large scale soil survey^46^. The divergence in bacterial community is probably due to the adaptation of microorganisms living in these environments to the contrasting characteristics observed among five sites. As showed in Fig. 4, some genera whose relative abundances were positively related to EC, K^+^, Na^+^, Mg^2+^, Cl^−^ and SO_4_^2−^ were disappeared due to reclamation. Most of them such as *Gaetulibacter*, *Alcanivorax* and *Maribacter* are marine bacteria which were observed from the seawater, tidal flat and marine sediment^47–49^. Our result was consistent with the finding of Fu *et al*.^19^ who observed that five typical bacteria (*Gaetbulibacter*, *Sporosarcina*…) did not appear in reclamation soil. Obviously, the tideland reclamation, changing the near-marine environment to a terrestrial one, would drastically change the habitats of bacteria, and then these bacteria vanished progressively. In contrast, lots of genera such as *Niabella*, *Flavisolibacter* and *Xylophilus* were absent at tideland, but normally detected at reclaimed soils. Besides, their relative abundances were significantly positively correlated with MWD and TN, while negatively related to EC, K^+^, Na^+^, Mg^2+^, Cl^−^ and SO_4_^2−^. Taking the genus *Niabella* for example, so far, the species of with validly published names were isolated from soil and plant^50, 51^, and no representative strains have been cultured from their habitats. This genus comprises strictly aerobic that might explain why we didn’t observe *Niabella* at tideland. To the best of our knowledge, the function of the genus is still unclear. Here, we can demonstrate that large soil aggregates, high nutrition and low EC were ideal for the growth of *Niabella*. Our results, one side, could indicate that both anthropogenic activity of reclamation and its histories could greatly alter soil bacterial community structure; other side, may suggest that bacterial community include a reservoir of species with the ability to quickly respond to ecological processes, which arises with the environmental change^29, 52^.

## Conclusion

In this study, the progressive reclamation induced larger soil aggregates, higher nutrients and lower EC. Concomitantly, a consistent shift of bacterial community structure and an increase in bacterial diversity and richness (phylogenetic diversity, Shannon, Chao1 and observed species) from tideland to reclamation sites were detected. Rather than pH, a dominant factor MWD was observed for shaping bacterial community structure. These findings strongly indicate that coastal land reclamation has positive effects on soil ecosystem, and will contribute to a comprehensive understanding of the responses of bacterial community to different reclamation histories in coastal areas.

## Materials and Methods

### Site description and soil sampling

The research area (32° 38′ N – 32° 45′ N, 120° 53′ E – 120° 57′ E) was located in Dongtai City, Jiangsu Province, Eastern China. In this area, intertidal marsh flats have been reclaimed by constructing dikes for nearly1000 years. This area has a North Asia subtropics monsoon climate, with mean temperature 15.0 °C, average rainfall about 1061.2 mm, a total annual sunshine time of 2130.5 h and more than 220 frost-free days.

Five sampling sites were selected on Arp. 10, 2013, including a tideland and four sites reclaimed from natural tidal flats by constructing dikes in 2012, 2007, 1995 and 1975, respectively (represented as native, 2-year, 7-year, 19-year and 39-year lands, respectively). Native tideland was located to the east of 2012 dike, situated in the tidal marsh flat and was flooded during spring tides. It was bare, with primary producer microalgae. At 2-year site, the pioneer population of *Suaeda salsa* was established, with vegetation coverage of around 15%. 7-year site was covered by *S. salsa*, *Imperata cylindrica* and *Phragmites australis* and the total vegetation coverage >80%. 19-year and 39-year sites were cultivated agricultural land and planted with paddy rice, wheat, maize and so on. However, in this study, soil samples were collected in the fallow lands which were near the initial dikes. The dominant plants were *Capsella bursa-pastoris*, *Veronica persica* and *Descurainia sophia* at 19-year site and were *Populus L*., *Capsella bursa-pastoris* and *Euphorbia helioscopia* at 39-year site.

Soil samples were collected at 0 to 20 cm with a corer (2.5 cm in diameter), and three replicate cores on each sampling occasion were homogenized to create one sample for each site. Thus, a total of nine cores were sampled for each site. Subsamples were air-dried and sieved with nylon mesh for physical and chemical analysis. Others were stored at −80 °C for high throughput sequencing analyses.

### Soil physical and chemical analysis

Soil bulk density (BD) was measured using stainless steel ring and oven-dried at 105 °C. Water stable aggregate size distribution was carried out by wet-sieving the field-moist soil using a vibratory sieve shaker (AS 200 basic, Retsch, Haan, Germany). Briefly, a subsample of 50 g field-moist soil was passed through a 4-mm sieve by gently breaking soil clods along natural planes of fracture. Then, soil was fractionated through a series of four sieves as follows: 1.0, 0.25 and 0.053 mm. The resulting four sizes are large aggregates (>1.0 mm, LA), macroaggregates (1.0–0.25 mm, MAA), microaggregates (0.25–0.053 mm, MIA) and silt + clay fractions (<0.053 mm, SC). The aggregates retaining on each sieve and collecting pan were put on a weighted filter, which was oven-dried at 105 °C for 6 h and weighed to determine the proportion of whole soil weight in each fraction. Mean weight diameter of soil aggregates (MWD) was calculated using the following equation:

$$MWD=\sum _{i=1}^{n}{\bar{x}}_{i}\times {w}_{i}$$

where $\,{\bar{x}}_{i}\,$is the average diameter (mm) of each size and *w*_*i*_ is the proportion of the whole soil in this fraction.

Soil pH was determined with a glass electrode using a soil-to-water ratio of 1:2.5. Soil organic carbon was determined by dichromate oxidation and a constant 1.724 was used to convert organic carbon to organic matter (OM). Soil total nitrogen (TN) was determined by Kjeldahl digestion. Soil available phosphorus (AP) was tested using the molybdenum blue method. Electric conductivity (EC) was measured by an electric conductivity meter 873CC (FOX BOLO CO., LTD). Soil exchangeable ions, *i.e*. K^+^, Ca^2+^, Na^+^, Mg^2+^, and Cl^−^, SO_4_^2−^, HCO_3_^−^, CO_3_^2−^ were water-extracted using a soil-to-water ratio of 1:50 and measured by inductively coupled plasma atomic emission spectrometry (ICP-AES, IRIS Advantage, Thermo, USA) and ion chromatography (CS-1100, Thermo, USA), respectively. The accuracy of the analyses was estimated by comparison with a reference material GBW07413 from Institute of Geophysical & Geochemical Exploration, Chinese Academy of Geosciences, and blanks were introduced regularly.

### Soil DNA extraction and high throughput sequencing

Soil total DNA was extracted from 0.5 g of moist soil using a FastDNA® SPIN Kit for Soil (MP Biomedicals, Santa Ana, CA) according to manufacturer’s instructions. The extracted soil DNA was dissolved in 50 µl of TE buffer, quantified by a spectrophotometer and stored at −20 °C until further use. A total of 15 DNA samples were used for Quantitative PCR (qPCR) and high throughput sequencing analyses.

Soil bacterial 16 S rRNA gene was amplified by using the prime set of 519 F (CAGCMGCCGCGGTAATWC) and 907 R (CCGTCAATTCMTTTRAGTTT) with an average length of 400 bp^53^. To perform high throughput sequencing on the Illumina Miseq platform (Illumina, Inc., CA, USA), the oligonucleotide sequences included a 5-bp barcode fused to the forward primer as follows: barcode + forward primer. PCR was carried out in 50 µl reaction mixtures with the following components: 4 µl (initial 2.5 mM each) of deoxynucleoside triphosphates, 2 µl (initial 10 µM each) of forward and reverse primers, 2 U of *Taq* DNA polymerase with 0.4 µl (TaKaRa, Japan), and 1 µl of template containing approximately 50 ng of genomic community DNA as a template. Thirty-five cycles (95 °C for 45 s, 56 °C for 45 s, and 72 °C for 60 s) were performed with a final extension at 72 °C for 7 min. The products were then purified by using QIAquick PCR Purification kit (QIAGEN). After qualified by using Nanodrop ND-1000, all samples were normalized in equimolar amounts, and then prepared using TruSeq™ DNA Sample Prep LT Kit and sequenced using MiSeq Reagent Kit (500-cycles-PE) following the manufacturer’s protocols.

### Processing of the sequencing data

The bacterial 16 S rRNA gene data were processed using the Quantitative Insights Into Microbial Ecology (QIIME) as previously described^54^. Briefly, reads with an average quality score below 25 or shorter than 200 bp were discarded. Then, the sequences were denoised and binned into Operational Taxonomic Units (OTUs) using UCLUST based on a 97% identity threshold. The most abundant sequence from each OTU was selected as a representative sequence for that OUT according to PyNAST. Taxonomy was assigned to bacterial OTUs against a subset of the Silva 104 database. In order to compare the similarity between bacterial communities from soil samples, the diversity of each sample was estimated using Shannon index, Faith’s index of phylogenetic diversity, observed species and Chao 1.

### Statistical analysis

The data of soil properties and relative abundance of bacterial phyla were analyzed by a one-way analysis of variance (ANOVA) with treatment as factor by SPSS 18.0 for Windows. Mean separation was conducted based on Duncan’s multiple range test, and differences at *P* < 0.05 were considered statistically significant. Spearman’s correlation coefficient calculated by SPSS 18.0 for Windows was used to investigate the possible correlations between soil properties and bacterial diversity, relative abundance of bacterial phyla and some distinct groups. Non-metric multidimensional scaling (NMDS) analyses used for ordination based on the Unifrac phylogenetic distance matrix for bacterial community was performed by R software (Version 3.0.2, vegan package). Redundancy analysis (RDA) was calculated by Canoco version 4.5 to elucidate the relationships between soil microbial parameters and soil properties. Distance-based regression analysis was applied to identify variables that explained significant amounts of variation in bacterial community structure. The analysis was carried out using the DISTLM program with forward selection procedure and 9999 permutations. All data generated or analysed during this study are included in this published article (and its Supplementary Information files).

## Electronic supplementary material

Supplementary information
