# Supplementary material for: Shift of bacterial community structure along different coastal reclamation histories in Jiangsu, Eastern China
Source: Sci Rep. 2017 Aug 30;7:10096. doi: 10.1038/s41598-017-10608-3 (PMC5577240; doi:10.1038/s41598-017-10608-3)
Supplement: Supplementary file 1 — Supplementary information [file 41598_2017_10608_MOESM1_ESM.pdf]

Shift of bacterial community structure along different coastal reclamation histories in Jiangsu, Eastern China

Jianfeng Hua[1,2], Youzhi Feng[2], Qian Jiang[2], Xuewen Bao[3] & Yunlong Yin[1]

[1]Institute of Botany, Jiangsu Province and Chinese Academy of Sciences, Nanjing China. [2]State Key Laboratory of Soil and Sustainable Agriculture, Institute of Soil Science, Chinese Academy of Sciences, Nanjing China. [3]Nanjing Forest Police College, Nanjing China.

	BD	MWD	LA	MAA	MIA	SC	pH	TN	OM	C:N	AP	EC	Ca^{2+}	K^+	Mg^{2+}	Na^+	Cl^-	SO_4^{2-}	CO_3^{2-}	HCO_3^-
Bacteiral diversity and richness																				
Observed species	-0.24	0.779**	0.725**	0.663**	0.793**	-0.811**	0.086	0.806**	0.672**	-0.665**	0.038	-0.808**	-0.596*	-0.668**	-0.843**	-0.754**	-0.775**	-0.793**	-0.284	0.639*
Chao1	-0.132	0.790**	0.737**	0.606*	0.843**	-0.839**	0.154	0.857**	0.731**	-0.643**	0.07	-0.794**	-0.618*	-0.639*	-0.814**	-0.746**	-0.779**	-0.775**	-0.303	0.664**
PD	-0.335	0.820**	0.790**	0.713**	0.821**	-0.850**	0.077	0.819**	0.699**	-0.606*	0.054	-0.865**	-0.596*	-0.682**	-0.886**	-0.829**	-0.846**	-0.857**	-0.288	0.621*
Shanon	-0.269	0.803**	0.721**	0.710**	0.789**	-0.821**	0.073	0.757**	0.640*	-0.606*	0.002	-0.810**	-0.571*	-0.686**	-0.850**	-0.757**	-0.771**	-0.789**	-0.234	0.625*
Relative abundance of taxonomy																				
Acidobacteria	-0.448	0.849**	0.810**	0.806**	0.796**	-0.811**	-0.004	0.844**	0.710**	-0.629*	0.050	-0.922**	-0.582*	-0.729**	-0.918**	-0.954**	-0.964**	-0.954**	-0.313	0.568*
Actinobacteria	-0.861**	0.588*	0.604*	0.747**	0.518*	-0.568*	-0.340	0.437	0.420	-0.298	-0.154	-0.626*	0.011	-0.443	-0.632*	-0.650**	-0.564*	-0.636*	-0.456	-0.079
Bacteroidetes	0.426	-0.718**	-0.564*	-0.788**	-0.671**	0.714**	0.032	-0.577*	-0.484	0.631*	0.150	0.795**	0.354	0.679**	0.804**	0.786**	0.718**	0.768**	0.294	-0.296
Chloroflexi	-0.317	0.670**	0.475	0.740**	0.625*	-0.679**	-0.018	0.343	0.352	-0.427	-0.336	-0.715**	-0.154	-0.729**	-0.686**	-0.661**	-0.575*	-0.625*	-0.232	0.164
Cyanobacteria	-0.095	-0.422	-0.073	-0.427	-0.371	0.411	-0.566*	-0.254	0.036	0.561*	0.290	0.458	0.864**	0.518*	0.486	0.489	0.489	0.493	-0.613*	-0.789**
Gemmatimonadetes	-0.358	0.651**	0.347	0.720**	0.600*	-0.632*	0.215	0.254	0.089	-0.640*	-0.356	-0.690**	-0.396	-0.682**	-0.664**	-0.661**	-0.600*	-0.657**	0.079	0.254
Nitrospirae	-0.324	0.799**	0.862**	0.642**	0.818**	-0.814**	0.057	0.898**	0.774**	-0.577*	0.161	-0.903**	-0.657**	-0.682**	-0.879**	-0.900**	-0.943**	-0.918**	-0.331	0.679**
Planctomycetes	0.415	-0.084	-0.447	0.002	-0.157	0.111	0.449	-0.327	-0.475	-0.375	-0.486	-0.102	-0.507	-0.368	-0.114	-0.071	-0.014	-0.075	0.688**	0.454
Verrucomicrobia	0.591*	-0.763**	-0.717**	-0.752**	-0.721**	0.743**	-0.048	-0.672**	-0.515*	0.499	-0.063	0.781**	0.514*	0.532*	0.789**	0.829**	0.836**	0.832**	0.173	-0.414
Alphaproteobacteria	0.562**	-0.618*	-0.830**	-0.531*	-0.682**	0.654**	0.199	-0.797**	-0.718**	0.320	-0.250	0.593*	0.218	0.350	0.561*	0.600*	0.646**	0.618*	0.549*	-0.250
Betaproteobacteria	-0.394	0.685**	0.854**	0.509	0.700**	-0.689**	-0.113	0.797**	0.781**	-0.393	0.113	-0.799**	-0.357	-0.596*	-0.754**	-0.779**	-0.814**	-0.807**	-0.543*	0.414
Deltaproteobacteria	0.725**	-0.699**	-0.685**	-0.660**	-0.732**	0.754**	-0.030	-0.565*	-0.425	0.470	0.118	0.636**	0.221	0.421	0.636*	0.596*	0.586*	0.650**	0.333	-0.114
Gammaproteobacteria	0.544*	-0.736**	-0.770**	-0.667**	-0.707**	0.721**	0.149	-0.712**	-0.733**	0.318	0.154	0.717**	0.143	0.600*	0.746*	0.721**	0.675**	0.700**	0.561*	-0.214

Supplementary Table S1. The spearman's correlations between bacterial community and soil properties. PD, phylogenetic diversity index; BD, soil bulk density; MWD, mean weight diameter of soil aggregates; LA, large aggregates (> 1.0 mm); MAA, macroaggregates (1.0–0.25 mm); MIA, microaggregates (0.25–0.053 mm); SC, silt + clay fractions (< 0.053 mm); TN, total nitrogen; OM, organic matter; AP, available phosphorus; EC, electric conductivity. $^{**}P < 0.01$ and $^{*}P < 0.05$ indicate significant correlations.

	BD	MWD	LA	MAA	MIA	SC	pH	TN	OM	C: N	AP	EC	Ca^{2+}	K^{+}	Mg^{2+}	Na^{+}	Cl^{-}	SO_4^{2-}	CO_3^{2-}	HCO_3^{-}
Genera whose relative abundances were decreased with increasing reclamation histories																				
Endosymbionts	0.501	-0.881**	-0.843**	-0.705**	-0.891**	0.898**	-0.128	-0.838**	-0.709**	0.583*	0.106	0.847**	0.491	0.710**	0.839**	0.802**	0.828**	0.847**	0.344	-0.522*
Acidithiobacillus	0.618*	-0.952**	-0.843**	-0.823**	-0.953**	0.953**	-0.124	-0.815**	-0.642**	0.650**	0.095	0.895**	0.572*	0.758**	0.883**	0.865**	0.887**	0.894**	0.229	-0.570*
Desulfuromusa	0.633*	-0.941**	-0.843**	-0.860**	-0.931**	0.939**	-0.048	-0.868**	-0.705**	0.617*	0.056	0.873**	0.531*	0.736**	0.865**	0.865**	0.876**	0.872**	0.275	-0.559*
Balneola	0.599*	-0.892**	-0.843**	-0.783**	-0.850**	0.876**	-0.113	-0.823**	-0.698**	0.622*	0.051	0.951**	0.605*	0.813**	0.953**	0.931**	0.946**	0.950**	0.273	-0.629*
Maribacter	0.588*	-0.897**	-0.843**	-0.772**	-0.876**	0.883**	-0.087	-0.847**	-0.720**	0.661**	0.023	0.962**	0.642**	0.813**	0.965**	0.946**	0.961**	0.961**	0.290	-0.662**
Kangiella	0.559*	-0.838**	-0.843**	-0.698**	-0.854**	0.869**	-0.046	-0.761**	-0.635*	0.565*	0.134	0.836**	0.420	0.688**	0.810**	0.769**	0.791**	0.835**	0.365	-0.433
Gaetbulibacter	0.674**	-0.936**	-0.843**	-0.871**	-0.931**	0.935**	0.000	-0.840**	-0.694**	0.613*	0.077	0.917**	0.572*	0.773**	0.902**	0.917**	0.917**	0.917**	0.260	-0.596*
Alcanivorax	0.490	-0.844**	-0.843**	-0.661**	-0.847**	0.861**	-0.128	-0.785**	-0.683**	0.532*	0.114	0.840**	0.446	0.695**	0.832**	0.788**	0.813**	0.839**	0.365	-0.470
Brumimimicrobium	0.605*	-0.886**	-0.843**	-0.768**	-0.865**	0.894**	-0.113	-0.737**	-0.604*	0.598*	0.139	0.917**	0.527*	0.780**	0.898**	0.861**	0.883**	0.917**	0.258	-0.529*
Genera whose relative abundance were increased with increasing reclamation histories																				
Variovorax	-0.670**	0.862**	0.849**	0.751**	0.828**	-0.835**	0.047	0.892**	0.753**	-0.604*	0.011	-0.897**	-0.601*	-0.705**	-0.913**	-0.918**	-0.942**	-0.939**	-0.329	0.610*
Flavisolibacter	-0.738**	0.861**	0.752**	0.866**	0.821**	-0.857**	-0.066	0.679**	0.504	-0.456	-0.265	-0.830**	-0.272	-0.703**	-0.821**	-0.810**	-0.756**	-0.810**	-0.296	0.240
Xylophilus	-0.532*	0.940**	0.837**	0.832**	0.953**	-0.964**	0.081	0.802**	0.662**	-0.567*	-0.084	-0.861**	-0.548*	-0.746**	-0.857**	-0.821**	-0.839**	-0.839**	-0.215	0.602*
Polaromonas	-0.683**	0.815**	0.891**	0.772**	0.782**	-0.794**	-0.283	0.831**	0.823**	-0.387	0.042	-0.800**	-0.238	-0.564*	-0.850**	-0.822**	-0.794**	-0.814**	-0.614	0.278
Niabella	-0.604*	0.918**	0.655**	0.863**	0.871**	-0.903**	0.212	0.649**	0.490	-0.705**	-0.407	-0.917**	-0.573*	-0.918**	-0.907**	-0.878**	-0.839**	-0.885**	-0.083	0.577*
Aquamonas	-0.515*	0.878**	0.846**	0.779**	0.834**	-0.859**	0.058	0.750**	0.619*	-0.578*	0.014	-0.849**	-0.405	-0.694**	-0.802**	-0.796**	-0.859**	-0.861**	-0.322	0.407
Gemmatimonas	-0.628*	0.951**	0.772**	0.879**	0.928**	-0.950**	0.183	0.727**	0.578**	-0.710**	-0.262	-0.967**	-0.563*	-0.892**	-0.953**	-0.932**	-0.910**	-0.939**	-0.204	0.545*
Methylibium	-0.611*	0.866**	0.795**	0.729**	0.876**	-0.894**	0.125	0.855**	0.720**	-0.670**	-0.138	-0.943**	-0.594*	-0.771**	-0.928**	-0.922**	-0.902**	-0.939**	-0.329	0.577*

Supplementary Table S2. The spearman's correlations between distinct bacterial genera and soil properties. BD, soil bulk density; MWD, mean weight diameter of soil aggregates; LA, large aggregates (> 1.0 mm); MAA, macroaggregates (1.0–0.25 mm); MIA, microaggregates (0.25–0.053 mm); SC, silt + clay fractions (< 0.053 mm); TN, total nitrogen; OM, organic matter; AP, available phosphorus; EC, electric conductivity. $^{**}P < 0.01$ and $^{*}P < 0.05$ indicate significant correlations.

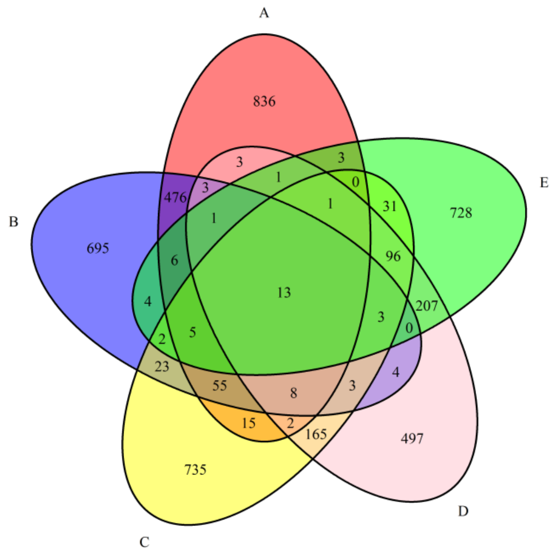

Supplementary Figure S1. Venn diagram showing overall overlap of operational taxonomic units (OTUs) between the five soils. OTUs are defined at 97% sequence similarity level. A, 39-year; B, 19-year; C, 7-year; D, 2-year; E, Native.
